# Chuankezhi injection for asthma: Protocol of a systematic review and meta-analysis

**DOI:** 10.1097/MD.0000000000016630

**Published:** 2019-08-16

**Authors:** Meichen Li, Wenjiang Zheng, Chaoyuan Zhang, Qian Yan, Zijing Peng, Fuqi Xie, Yu Hong, Xiaohong Liu

**Affiliations:** a Clinical Medical College of Acupuncture Moxibustion and Rehabilitation; b Guangzhou University of Chinese Medicine; c The First Affiliated Hospital of Guangzhou University of Chinese Medicine, Guangzhou, China.

**Keywords:** asthma, chuankezhi injection, meta-analysis, protocol, systematic review

## Abstract

**Background:**

Asthma is a chronic inflammatory disease characterized by recurrent attacks of breathlessness and wheezing, which often worsen at night or in the early morning and vary from person to person in severity and frequency. Chuankezhi injection (CKZ), as a new Chinese medicine, was recently found to have a good clinical effect on asthma. Whereas neither systematic nor meta-analysis of randomized controlled trials (RCTs) explain the efficacy of CKZ in treating asthma. Therefore, we provide a protocol to evaluate the efficacy and safety of CKZ for asthma.

**Methods:**

From inception until April 2019, a systematic and comprehensive literature search will be conducted in both 4 Chinese databases and 3 English databases. RCTs will be included related to CKZ for asthma. We will assess the quality of the included trials in accordance with the risk of bias tools in Cochrane manual 5.1.0. We will use the Grading of Recommendations Assessment, Development, and Evaluation (GRADE) method to assess the certainty of the estimated evidence. Data analysis will be performed using the STATA 15.0.

**Results:**

This systematic review aims to assess the effectiveness and safety of CKZ for the treatment of asthma, in order to provide evidence for the clinical practice of Chinese medicine. This protocol will be conducted and reported according to the Preferred Reporting Items for Systematic Reviews and Meta-Analyses Protocols (PRISMA-P) statement. The results of this meta-analysis will be submitted to a peer-reviewed journal once it is completed.

**Conclusion:**

The consequence of this study will furnish proof to evaluate if CKZ is effective in the treatment of asthma.

**PROSPERO registration number:**

ROSPERO CRD42019134458.

## 1 Introduction

Asthma is a highly prevalent chronic inflammatory airway disease characterized clinically by repeated episodes of wheezing, breathlessness, chest tightness, and coughing, usually in the presence of variable (and reversible) airflow obstruction.^[1]^ Over the past 20 years, the basic understanding of asthma and its pathogenesis has rapidly evolved. Immunologically related mechanisms are prominent in asthma susceptibility leading to the development of novel pharmacological therapies.^[2]^ Conventional treatments include inhaled corticosteroids, long-acting 2 agonists (LABAs), agents that affect the leukotriene pathway, combination products, and monoclonal anti-IgE therapies.^[3,4,5]^ However, these have been proved to represent either limited efficacy or frequent side effects, for example, mood changes, transient effects, immunosuppression, side-effects of steroids and even increasing the risk of asthma-related death and hospitalization.^[6,7,8,9]^ Many patients with asthma have uncontrolled disease despite treatment with inhaled glucocorticoid.^[10]^ Therefore, US guidelines suggest a stepwise approach to modifying therapy.^[11]^

Chuankezhi injection (Chinese herb injection) was recently found to play an important role in relieving cough and asthma and to have anti-allergy, anti-inflammatory, stress response, and immunoregulatory functions. Peripheral blood T lymphocytes of asthmatic patients who were treated with Chuankezhi injection (CKZ) secreted more interferon-gamma (IFN-γ) and less interleukin-4 (IL-4).^[12,13,14]^ With the publication of a number of trials on CKZ for asthma, they have certificated that CKZ has a good clinical effect. There is an urgent need for a systematic review to support the effectiveness and safety of CKZ in treating asthma. Hereby, the purpose of the study is to systematically review current available randomized controlled trials (RCTs) to assess the efficacy and safety of the CKZ treatment in patients of asthma.

## 2 Methods

### 2.1 Study registering and reporting

The protocol has been registered on PROSPERO (ID CRD42019134458) basing on the Preferred Reporting Items for Systematic Reviews and Meta-Analyses Protocols (PRISMA-P) statement guidelines.^[15]^

### 2.2 Eligibility criteria

The eligibility criteria for the review using the PICOS (population-intervention-comparative-results-study design) framework are as follows.

#### 2.2.1 Population

According to the GINA diagnostic criteria,^[1]^ this study will include patients (adults over 18 years old) who meet the diagnosis of asthma irrespective of their sex or ethnicity. Patients with other complicating diseases will not be included. Even the patients’ course of disease and severity of illness will be approximately equivalent.

#### 2.2.2 Interventions/Comparators

In the treatment group, CKZ combined with Western medicine or CKZ alone, while the controls could be no intervention, placebo, or western medication.

#### 2.2.3 Outcome measures

The following primary outcomes will be measured: number of severe exacerbations during the invention or in a follow-up; forced expiratory volume in 1 second (FEV_1_); forced vital capacity (FVC); forced expiratory volume in 1 second/forced vital capacity (FEV_1_/FVC); total effective rate; peak expiratory flow (PEF); adverse effects; the Asthma Control Test or the 5-item Asthma Control Questionnaire (ACQ-5); the Asthma Quality of Life Questionnaire.

#### 2.2.4 Study design

Regardless of the blind method and language, only RCTs will be included. The following types of studies will be excluded: nonclinical researches; duplicate publications; literature lacking data that are needed for our study; studies that have no diagnostic criteria or efficacy criteria; and the baseline data are significantly inconsistent.

### 2.3 Search strategy

We will start from the database by searching 4 Chinese databases (China national knowledge infrastructure database, wan fang database, Chongqing VIP information, and SinoMed) and 3 English databases (Medline, Cochrane library, and Embase). In order to obtain the potential nonelectronic literature, relevant magazines and medical journals will also be filtered for further search. In these databases, strict restrictions will be placed to exclude the types of studies that are not RCTs. During the searches, there will be no restrictions on languages. However, once the search has been conducted, papers that are not in English or Chinese will be excluded. The search strategy of Medline is as follows:

#1 Search (“Asthma”[Mesh]) OR ((“Asthmas” or “Bronchial Asthma” or “Asthma, Bronchia ”)).
#2 Search ((“Chuankezhi” or “ Chuankezhi injections” or “ Chuankezhi injection” or “ Chuankezhi injectables ” or “ Chuankezhi injectable”)).
#3 #1 AND #2.

### 2.4 Study selection and data extraction

#### 2.4.1 Selection of studies

After removing duplicates, 2 reviewers (QY and CZ) will independently evaluate the titles and abstracts of the searched studies by EndNote X9.0 (Stanford, Connecticut, https://endnote.com). Independently screen the included studies, extract data, evaluate the quality of included studies and cross-check with each other according to the established selection criteria. Any different opinions generated between the 2 reviewers will be resolved through discussion. When consultation fails to reach an agreement, the third reviewer (WZ) will step in and provide arbitration. A PRISMA flow chart will be designed to illustrate the study selection procedure.

#### 2.4.2 Data extraction and management

We will check the final results of the data extraction and provide arbitration for further disagreements. Microsoft Excel (Redmond, Washington, https://www.microsoft.com/zh-cn) will be used to extract data and collect relevant information. We extracted and recorded the first author's name, year of publication, study design, intervention, and control group information, sample size, duration of intervention, and outcomes, including number of severe exacerbations during the invention or in a follow-up, FEV_1_, FVC, FEV_1_/FVC, total effective rate, PEF, adverse effects, etc. We will contact the corresponding authors for additional information if necessary.

### 2.5 Quality assessment

The risk of bias tool (ROB) in Cochrane Handbook 5.1.0 will be used to assess the methodological quality. Seven items are included: random sequence generation, allocation concealment, blinding of participants and personnel, blinding of outcome assessment, incomplete outcome data, selective reporting, and other sources of bias. The judgment of each item is divided into 3 levels: low risk of bias, high risk of bias, and unclear risk of bias. Sensitivity analysis will also be conducted excluding studies reporting the high risk of bias in any domain analyzed. To grade evidence quality and understand the current situation of evidence rating thereby analyzing possible problems, We will assess The Grading of Recommendations Assessment, Development and Evaluation (GRADE) system to grade the evidence quality of published systematic reviews/meta-analyses of asthma.^[16]^ Based on 5 downgrade factors (bias, indirection, inconsistency, imprecision, and risk of publication bias), the quality of evidence is divided into 1 of 4 levels: high, medium, low, and very low.

### 2.6 Statistical analysis

For continuous variables, we will use the weighted mean difference (WMD) to evaluate the extracted data. For dichotomous variables, the odds ratio (OR) will be applied to analyze. The confidence intervals (CI) for both continuous and dichotomous variables will be set to 95%.

The heterogeneity test will be performed by using STATA 15.0 software (StataCorp, College Station, TX, https://www.stata.com). Statistical heterogeneity was statistically computed by using the chi-squared test and the inconsistency index statistic. If *I* ^2^ ≤ 50% and *P *≥* *.05, it suggests that heterogeneity is not important and the Mantel-Haenszel fixed model will be employed for meta-analysis. If *I* ^2^ > 50% and *P* < .05, it manifests that heterogeneity needs to be analyzed. Subgroup analysis, meta-regression, or descriptive analysis will be used for heterogeneity analysis.^[17]^ There are 3 kinds of heterogeneity—statistical heterogeneity, clinical heterogeneity, and methodological heterogeneity.^[18]^ The random effects model will be used for statistical heterogeneity. Subgroup analysis or meta-regression analysis will be performed if clinical and methodological heterogeneity exists. In addition, potential sources of substantial heterogeneity were evaluated by sensitivity analysis. And publication bias was estimated by funnel plots.^[19]^ Meanwhile, to understand the current situation of evidence rating and analyze possible problems, the GRADE system was performed to grade the evidence quality of published systematic reviews/meta-analyses of asthma.

### 2.7 Patient and public involvement

This part is not covered in this study.

## 3 Discussion

An important concept in asthma is that mechanisms of disease that originate in the lung drive airway pathology and airway dysfunction and explain the marked heterogeneity in phenotypes. Unfortunately, efforts so far to uncover new treatments to asthma have not been very productive.^[20]^ Traditional Chinese medicine (TCM) has a unique advantage in relieving and curing asthma because of its good curative effect and fewer adverse reactions. There are a number of anti-asthma herbal formulas recorded TCM literatures and used in practice, but evidence-based researches into their efficacy and mechanisms of efficacy are still in their infancy.^[21]^ CKZ is a new Chinese medicine that is primarily composed of extracts of *Morinda officinalis* and Epimedium. Icariin which is a flavonoid glucoside isolated from *Epimedium brevicornu* Maxim, has been reported to have anti-osteoporotic, anti-inflammatory, and antidepressant-like activities.^[22]^ *Morinda officinalis* var. *officinalis* is the most famous one among all the Chinese species, as its dried roots have long been used as 1 of the well-known 4 “Southern herbs” in traditional Chinese medicine, with the efficacy of invigorating kidney, strengthening muscle and bone, consolidating physical strength, and so on.^[23]^ CKZ was recently found to play an important role in relieving cough and asthma and to have anti-allergy, anti-inflammatory, stress response, and immunoregulatory functions.^[14]^ By stimulating the hypothalamic-pituitary-adrenal cortex axis, it can release hormones, improve the patient's immune function, reduce the dependence on adrenocortical hormone and ultimately improve the body's immunity.^[24,25,26]^

This systematic review aims to assess the effectiveness and safety of CKZ for the treatment of asthma, in order to provide evidence for the clinical practice of Chinese medicine. We should pay specific attention to 2 issues in this study. Firstly, we have found that randomization was not designed rigorously in most studies published in Chinese journals based on our experience. Predictably, most of the randomized controlled trials included in this study will be rated as low-level quality.^[15]^ Secondly, this study is based on literature analysis. The presence of heterogeneity is an inherent problem in a meta-analysis, which would affect the estimate. It results from the diversity in clinical and methodological characteristics, and variations between studies.^[18]^ As this study is secondary research based on literature, ethics approval and patient consent are not necessary. This protocol will be conducted and reported according to the PRISMA-P statement. The results of this meta-analysis will be submitted to a peer-reviewed journal once it is completed.

## Author contributions

**Conceptualization:** Meichen Li, Wen Jiang Zheng.

**Data curation:** Chaoyuan Zhang, Qian Yan.

**Investigation:** Wen Jiang Zheng, Zijing Peng, Fuqi Xie, Yu Hong.

**Methodology:** Meichen Li, Wen Jiang Zheng, Chaoyuan Zhang.

**Project administration:** Wen Jiang Zheng, Xiaohong Liu.

**Resources:** Wen Jiang Zheng, Yu Hong.

**Software:** Chaoyuan Zhang.

**Supervision:** Xiaohong Liu.

**Writing – original draft:** Meichen Li, Wen Jiang Zheng, Qian Yan, Zijing Peng, Fuqi Xie.

**Writing – review & editing:** Meichen Li, Wen Jiang Zheng, Xiaohong Liu.

Wenjiang Zheng orcid: 0000-0001-5234-0339.

Xiaohong Liu orcid: 0000-0003-4795-3039.
